# DesignMaster: a multi-conditional diffusion framework for rational PROTAC design

**DOI:** 10.1093/bioinformatics/btag714

**Published:** 2026-09-24

**Authors:** Binze Shi, Yi Hao, Jie Liu, Tong Pan, Luke Isbel, Michael Roy, Ashley P Ng, Xuequn Shang, Fuyi Li

**Affiliations:** School of Computer Science, Northwestern Polytechnical University, Xi’an, Shaanxi 710129, China; South Australian immunoGENomics Cancer Institute (SAiGENCI), Adelaide University, Adelaide, SA 5005, Australia; College of Information Engineering, Northwest A&F University, Yangling, Shaanxi 712100, Australia; South Australian immunoGENomics Cancer Institute (SAiGENCI), Adelaide University, Adelaide, SA 5005, Australia; South Australian immunoGENomics Cancer Institute (SAiGENCI), Adelaide University, Adelaide, SA 5005, Australia; South Australian immunoGENomics Cancer Institute (SAiGENCI), Adelaide University, Adelaide, SA 5005, Australia; Adelaide Centre for Epigenetics (ACE), Adelaide University, Adelaide, SA 5005, Australia; South Australian immunoGENomics Cancer Institute (SAiGENCI), Adelaide University, Adelaide, SA 5005, Australia; Walter and Eliza Hall Institute of Medical Research, Parkville, VIC 3050, Australia; Sir Peter MacCallum Department of Oncology, The University of Melbourne, Parkville, VIC 3010, Australia; School of Computer Science, Northwestern Polytechnical University, Xi’an, Shaanxi 710129, China; South Australian immunoGENomics Cancer Institute (SAiGENCI), Adelaide University, Adelaide, SA 5005, Australia; College of Information Engineering, Northwest A&F University, Yangling, Shaanxi 712100, Australia; Sir Peter MacCallum Department of Oncology, The University of Melbourne, Parkville, VIC 3010, Australia; Department of Microbiology and Immunology, The University of Melbourne, Parkville, VIC 3010, Australia

## Abstract

**Motivation:**

Proteolysis-targeting chimeras (PROTACs) enable targeted protein degradation through ternary complex formation with E3 ubiquitin ligase. However, the rational design of PROTACs remains highly challenging due to limited structure–activity relationship data and the vast conformational diversity of linkers. Existing computational approaches can be broadly divided into structure-based ternary modelling methods and fragment-based linker generation models. Although these approaches have advanced PROTAC design, they typically neglect key physicochemical constraints and linker-length control during the generation process, causing the generated PROTACs to lack balanced structural properties required for effective ternary complex formation with drug-like characteristics.

**Results:**

To address these limitations, we propose DesignMaster, a diffusion-based generative framework that explicitly incorporates linker length and physicochemical properties as controllable conditioning signals. DesignMaster employs an E(3)-equivariant graph Transformer with a gated multi-condition fusion module to inject linker length and physicochemical constraints throughout the diffusion process, enabling fine-grained and constraint-aware molecular generation. Experiments on PROTAC-DB 2.0 and 3.0 demonstrate that DesignMaster achieves the best or highly competitive performance across Validity, Uniqueness, and Recovery. The Case study further shows that DesignMaster consistently achieves improved geometric agreement with the reference PROTAC conformations across both the 6W7O and 6HAY complexes, highlighting its potential for practical structure-guided and property-aware PROTAC design.

**Availability:**

The source code and datasets are available at https://github.com/ABILiLab/DesignMaster.

## 1 Introduction

Proteolysis-targeting chimeras (PROTACs), first introduced by Sakamoto *et al.* (2001), have emerged as a transformative modality for targeted protein degradation (Burslem and Crews 2020, Zheng *et al.* 2022). A PROTAC is a heterobifunctional molecule composed of three modular components: a ligand that binds a protein of interest (POI), a linker, and a ligand that recruits an E3 ubiquitin ligase (Békés *et al.* 2022, Li *et al.* 2022a, Zhong *et al.* 2024). By simultaneously engaging the POI and the E3 ligase, PROTACs promote formation of a ternary POI–PROTAC–E3 complex, thereby hijacking the cellular ubiquitin–proteasome system to induce polyubiquitination and subsequent degradation of the target protein (Arnold 2024, Liu *et al.* 2025). Distinct from conventional occupancy-driven inhibitors, PROTACs function through an event-driven and catalytic mechanism, enabling a single degrader molecule to participate in multiple rounds of target degradation at relatively low cellular concentrations. This makes PROTACs a highly promising strategy in anticancer drug development, particularly for targeting previously intractable cancer-associated proteins (Li *et al.* 2022b, Zhao and Dekker 2022).

The rational design of PROTACs has remained inherently challenging since their initial inception, owing to their synthetic and mechanistic complexity and the limited availability of detailed structure–activity and experimental data, particularly in the early development of the field. (Tran *et al.* 2022, Zheng *et al.* 2022). Moreover, the degradation efficiency of a given PROTAC is critically governed by the geometry and dynamics of the ternary complex, which are strongly influenced by linker length, composition, and flexibility (Paiva and Crews 2019). The vast conformational diversity arising from different linker designs, together with the many possibilities for POI/E3 binders and exit vectors, makes rational PROTAC optimisation a time-consuming and chemistry-intensive task, particularly in the absence of experimental structural data. Efficiently exploring linker designs remains a central bottleneck in PROTAC development and motivates the need for systematic, structure-aware computational generation (Krieger *et al.* 2023).

In recent years, various computational methods have emerged to support rational PROTAC generation, in part building on a limited but growing literature of structural and biophysical datasets. These methods can be broadly categorised into several complementary directions, including structure-based ternary complex modelling, fragment-based linker generation, with recent studies further incorporating constraint-aware generation and post-generation optimization strategies (Li *et al.* 2024). The first class focuses on explicitly constructing and evaluating the POI–PROTAC–E3 ternary complex using molecular docking (Nori *et al.* 2022, Zheng *et al.* 2022), molecular dynamics (MD) simulations (Ma *et al.* 2025), or scoring function-based protocols (Bai *et al.* 2021, Weng *et al.* 2021). While such physics-driven approaches provide mechanistic interpretability and valuable structural insight, they are computationally intensive and often lack standardised protocols for reliable ternary modelling. These limitations have motivated the development of a second class of methods that recast PROTAC design as a fragment-connection problem. In this case, the warhead and E3 ligand are treated as predefined fragments, and the central task is to generate an appropriate linker to connect them. For example, DeLinker (Imrie *et al.* 2020) and 3DLinker (Huang *et al.* 2022) employ graph generative models to construct chemically valid linkers based on the spatial arrangement between two fragments. DiffLinker (Igashov *et al.* 2024), DiffPROTACs (Li *et al.* 2024), and DiffSBDD Schneuing *et al.* (2024) leverage diffusion-based 3D generative modelling to generate linkers that are geometrically consistent between fragment anchor positions. Recent studies have introduced additional structural and chemical considerations into PROTAC design, such as PROTAC-INVENT (Li *et al.* 2023) with PTS-guided 3D linker generation and Pro-GAT (Vemuri *et al.* 2026) as a post-generation repair approach for chemically disconnected diffusion-generated candidates.

Despite these advances, existing PROTAC generation models remain limited in their ability to explicitly and jointly control key physicochemical properties and linker-length constraints during generation. Consequently, the sampled PROTACs may be geometrically and chemically valid yet unconstrained in properties such as molecular weight, polarity, or flexibility. Balancing binding and geometry for productive ternary complex formation (Roy *et al.* 2019) with physicochemical profiles to enable more favourable drug-like characteristics, including aqueous solubility, cell permeability, and stability, is therefore critical (Hornberger and Araujo 2023, Inganas *et al.* 2025). This lack of explicit control may ultimately limit the practical applicability of current generative approaches.

To address this, we propose DesignMaster, a diffusion-based generative framework that explicitly incorporates linker length and physicochemical properties as controllable conditioning signals. DesignMaster first represents PROTAC molecules using an E(3)-equivariant graph Transformer to capture their three-dimensional structural features. We then design a gated multi-condition fusion module to inject conditional information into every stage of the diffusion denoising trajectory, enabling fine-grained control over the generative process. Utilizing PROTAC-DB demonstrates state-of-the-art performance in terms of Validity, Uniqueness and Recovery. Furthermore, DesignMaster achieves a 33.85% decrease in root-mean-square deviation (RMSD) between the generated PROTAC and the ground-truth PROTAC BCPyr (PDB: 6W7O), demonstrating its effectiveness for practical structure-guided PROTAC development. A consistent advantage is also observed in the 6HAY complex, where DesignMaster achieves better geometric agreement and molecular similarity with the reference structure than DiffPROTACs. These results highlight the potential of DesignMaster as a practical computational tool to support controllable and property-aware PROTAC design.

## 2 Materials and methods

### 2.1 Data collection and preprocessing

Following Li *et al.* (2024), we conduct experiments on three complementary datasets: GEOM (Axelrod and Gomez-Bombarelli 2022), PROTAC-DB 2.0 (Weng *et al.* 2023), and PROTAC-DB 3.0 (Ge *et al.* 2025). GEOM provides large-scale 3D molecular conformations and is used to pretrain the E(3)-equivariant graph neural network (GNN) backbone, enabling robust geometric representation learning prior to fine-tuning on relatively small PROTAC datasets.

For downstream evaluation, we use PROTAC-DB 2.0 and the expanded PROTAC-DB 3.0 (Weng *et al.* 2023, Ge *et al.* 2025). Since PROTAC entries are provided in 2D format, we computationally generate 3D conformations and decompose each molecule into warhead, E3 ligand, and linker via graph-based substructure matching. Samples that cannot be reliably decomposed are removed.

Following prior work (Li *et al.* 2024), we adopt a consistent preprocessing protocol for both PROTAC-DB versions to ensure a fair comparison. Considering the dataset size and characteristics, the retained molecules are split into training, validation, and test sets at ratios of 5:1:1 for PROTAC-DB 2.0 and 8:1:1 for PROTAC-DB 3.0, respectively. Full preprocessing details are provided in Supplementary Section S1.

### 2.2 The framework of DesignMaster

An overview of the architecture of DesignMaster, and its key components are visualised in Fig. 1. DesignMaster is a diffusion-based generative model that explicitly incorporates linker length and physiochemical properties as controllable conditioning signals. It consists of four major components: diffusion network, denoising network, equivariant graph nerual network, and gated multi-condition fusion. These four components will be elaborated in the following sections.

**Figure 1:**
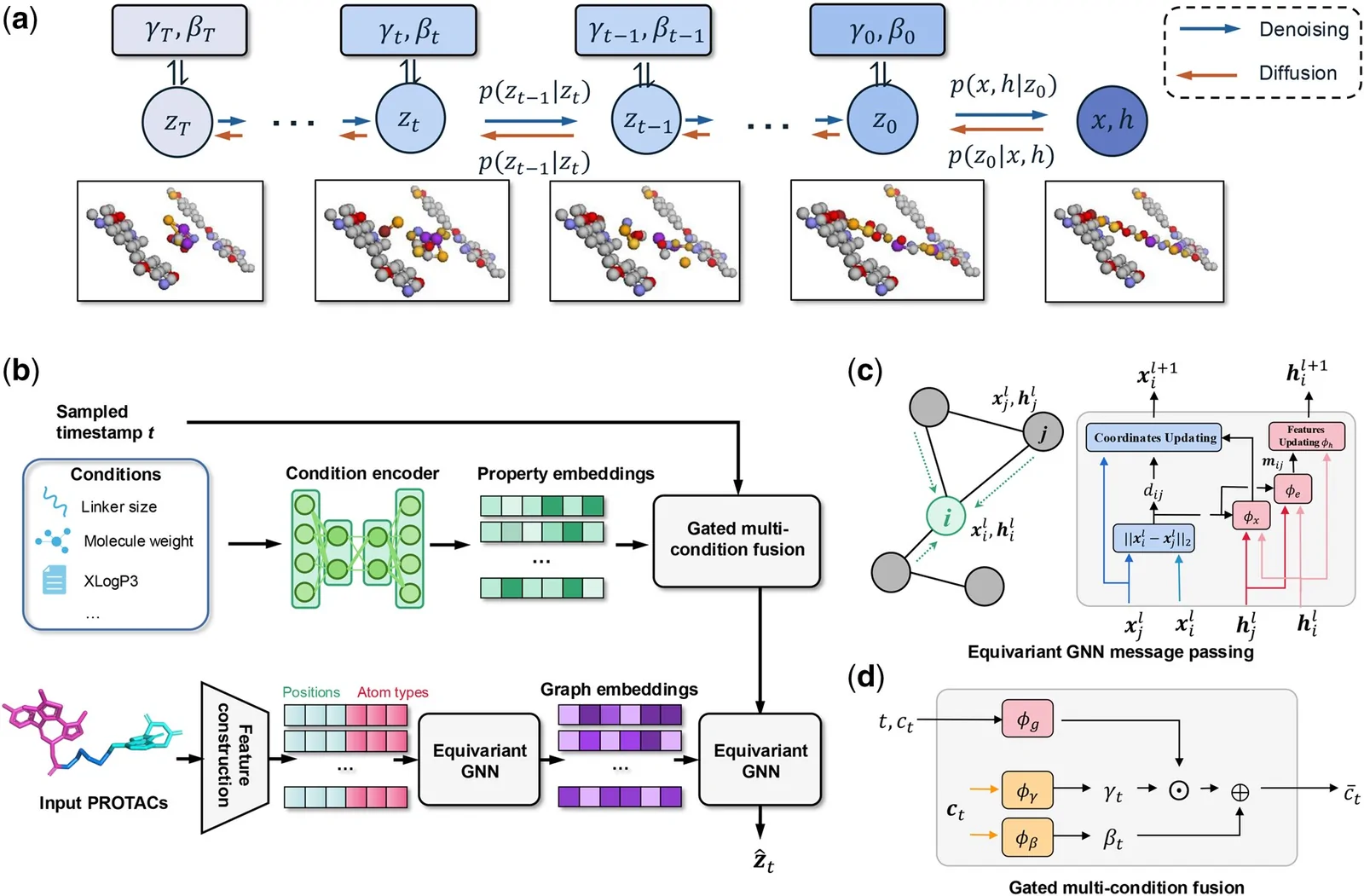
Overview of the DesignMaster framework. (a) Forward diffusion and reverse denoising process for conditional PROTAC generation, in which the warhead and E3 ligand remain fixed in both structure and spatial position, while the noisy latent variables $z_{t}$ are progressively denoised to synthesize the linker, resulting in the final molecule structure (x,h). (b) Overall model architecture. Molecular graphs and multi-property conditions (e.g. linker size, molecular weight, XLogP3) are encoded and fused via a gated multi-condition module to guide the equivariant generative process. (c) E(3)-equivariant GNN message passing, where atomic coordinates and features are jointly updated to preserve geometric equivariance. (d) Gated multi-condition fusion mechanism that modulates the diffusion dynamics by integrating conditional signals into the latent variables at each timestep.

#### 2.2.1 Diffusion process

We adopt a denoising diffusion probabilistic model (DDPM) to generate molecular structures in 3D space, following the formulation introduced by (Hoogeboom *et al.* 2022). As depicted in Fig. 1a, the model operates on a latent variable $z_{t}$, which represents the noisy molecular configuration at diffusion timestep *t*. Each $z_{t}$ consists of atomic coordinates $x_{t}\in R^{N\times 3}$ and associated node features $h_{t}\in R^{N\times nf}$, where *N* is the number of atoms and *nf* is the dimension of node features.

The forward diffusion process gradually perturbs the clean molecular representation $z_{0}$ by injecting Gaussian noise over *T* timesteps according to a predefined noise schedule ${\left\{\sigma_{t}^{2}\right\}}_{t=1}^{T}$:


(1) $$q(z_{t}|z_{t-1})=N\left(z_{t};\sqrt{1-\sigma_{t}^{2}}z_{t-1},\sigma_{t}^{2}I\right).$$

This process admits a closed-form marginal distribution:


(2) $$q(z_{t}|z_{0})=N\left(z_{t};\sqrt{{\bar{\alpha }}_{t}}z_{0},(1-{\bar{\alpha }}_{t})I\right),$$

where $\alpha_{t}=1-\sigma_{t}^{2},{\bar{\alpha }}_{t}=\prod_{s=1}^{t}\alpha_{s}$.

The reverse diffusion process aims to progressively denoise $z_{t}$ and recover a valid molecular structure conditioned on molecular context and external constraints. It is defined as a parameterized Gaussian transition:


(3) $$p_{\theta }(z_{t-1}|z_{t},u,c)=N\left(z_{t-1};\mu_{\theta }(z_{t},u,c_{t},t),{\tilde{\sigma }}_{t}^{2}I\right),$$

where ${\tilde{\sigma }}_{t}^{2}$ denotes the posterior variance. the mean is parameterized via a noise prediction network $\epsilon_{\theta }$:


(4) $$\mu_{\theta }(z_{t},u,c,t)=\frac{1}{\sqrt{\alpha_{t}}}\left(z_{t}-\frac{\sigma_{t}^{2}}{\sqrt{1-{\bar{\alpha }}_{t}}}\epsilon_{\theta }(z_{t},u,c_{t},t)\right).$$

Here, u is a binary mask that keeps the warhead and E3 ligand strictly fixed during generation, ensuring that only linker atoms are noised and updated while fragment coordinates and features are preserved at each denoising step. $c_{t}$ encodes additional conditioning information, specifically, $c_{t}$ encodes a 7-dimensional physicochemical property vector, including molecular weight (MW), hydrogen-bond acceptor count (HBA), hydrogen-bond donor count (HBD), XLogP3, rotatable-bond count (RB), heavy-atom count (HA), and topological polar surface area (TPSA) via learnable embeddings. During training, linker size is fixed by the molecular graph of each training example, while the continuous condition $c_{t}$ is constructed from the associated physicochemical annotations. At inference time, linker size is specified by the generation template, and $c_{t}$ may be provided as an external design constraint.

The denoising network is trained to predict the Gaussian noise added at each diffusion step. Specifically, a timestep t∼U{(1,…,T)} and noise ϵ∼N(0,I) are sampled, and the noisy input is constructed as:


(5) $$z_{t}=\sqrt{{\bar{\alpha }}_{t}}\;z_{0}+\sqrt{1-{\bar{\alpha }}_{t}}\;\epsilon .$$

The training objective is then defined as:


(6) $$L_{\text{diff}}=E_{z_{0},\;\epsilon ,\;t}\left[{\left\|\epsilon -\epsilon_{\theta }(z_{t},u,c_{t},t)\right\|}^{2}\right].$$

#### 2.2.2 Denoising network

We now describe the architecture used to parameterize the noise prediction function $\epsilon_{\theta }(z_{t},u,c,t)$. At diffusion timestep *t*, the noisy latent variable $z_{t}$ is decomposed into atomic coordinates and node features $z_{t}=(x_{t},h_{t})$. To extract geometry-aware representations, the pair $\left(x_{t},h_{t}\right)$ is first processed by an E(3)-equivariant graph neural network pretrained on the large-scale GEOM dataset:


(7) $$({\tilde{x}}_{t},{\tilde{h}}_{t})=f_{E3}^{\text{pre}}(x_{t},h_{t}).$$

This pretrained encoder serves as a geometry feature extractor, providing stable and physically meaningful representations of noisy molecular conformations. In parallel, the external condition encoding c is modulated by a time-dependent gated fusion mechanism to produce a timestep-aware conditioning signal:


(8) $${\tilde{c}}_{t}=f_{\text{fuser}}(c_{t},t),$$

where $f_{\text{fuser}}$ denotes the time-gated condition fuser. The fused condition features are then concatenated to the equivariant node features ${\hat{h}}_{t}=[{\tilde{h}}_{t}||{\tilde{c}}_{t}]$, while the coordinate representation $\tilde{x_{t}}$ is preserved to maintain equivariance. The augmented representations $\left(\tilde{x_{t}},{\hat{h}}_{t}\right)$ are subsequently passed to a second E(3)-equivariant GNN, which is trained from scratch and dedicated to denoising:


(9) $$({\bar{x}}_{t},{\bar{h}}_{t})=f_{E3}^{\text{denoise}}({\tilde{x}}_{t},{\hat{h}}_{t}).$$

Rather than directly predicting noise, the model outputs a denoised estimate of the molecular representation. The predicted noise is then obtained by computing the residual between the input and output representations:


(10) $$\epsilon_{\theta }(z_{t},u,c_{t},t)=\left(x_{t}-{\bar{x}}_{t},\;h_{t}-{\bar{h}}_{t}\right)⊙u.$$

This residual formulation aligns with standard DDPM training objectives and allows the model to focus on learning the denoising displacement while preserving E(3)-equivariance.

#### 2.2.3 Equivariant graph neural network

We adopt an E(3)-equivariant graph neural network to encode and denoise molecular structures in 3D space ($f_{E3}^{\text{pre}}(\cdot )$ and $f_{E3}^{\text{denoise}}(\cdot )$). Let $x_{t}\in R^{N\times 3}$ and $h_{t}\in R^{N\times nf}$ denote the coordinates and node features of atom *i*, respectively. Here, *nf* corresponds to the number of atom or molecular types, represented as a one-hot encoding. These features are subsequently projected into a higher-dimensional latent space within the network.

At layer ℓ pairwise messages are computed based on node features and relative geometry:


(11) $$m_{ij}^{\left(\ell \right)}=\phi_{e}\left(h_{i}^{\left(\ell \right)},h_{j}^{\left(\ell \right)},\|x_{i}^{\left(\ell \right)}-x_{j}^{\left(\ell \right)}{\|}^{2}\right),$$

where $\phi_{e}(\cdot )$ is a fully connected neural network shared across all edges. The coordinate update follows the standard equivariant formulation:


(12) $$x_{i}^{\left(\ell +1\right)}=x_{i}^{\left(\ell \right)}+\sum_{j\neq i}\frac{x_{i}^{\left(\ell \right)}-x_{j}^{\left(\ell \right)}}{\sqrt{d_{ij}^{\left(\ell \right)}+\epsilon }}\phi_{x}\left(m_{ij}^{\left(\ell \right)}\right),$$

where $d_{ij}^{\left(\ell \right)}={\left\|x_{i}^{\left(\ell \right)}-x_{j}^{\left(\ell \right)}\right\|}^{2}$, which ensures E(3)-equivariance by construction. The node features are updated by aggregating incoming messages:


(13) $$h_{i}^{\left(\ell +0.5\right)}=\sum_{j\neq i}m_{ij}^{\left(\ell \right)}.$$

To enhance the expressiveness of node feature updates, we implement $\phi_{h}(\cdot )$ using a Graphormer-style attention mechanism rather than a simple MLP. Given the aggregated message $h_{i}^{\left(\ell +0.5\right)}$, the node features are updated via self-attention over all nodes:


(14) $$h_{i}^{\left(\ell +1\right)}=\sum_{j}\alpha_{ij}^{\left(\ell \right)}W_{v}h_{j}^{\left(\ell +0.5\right)},$$

where $W_{v}$ is a learnable linear projection and $\alpha_{ij}^{\left(\ell \right)}$ denotes the attention weight between nodes *i* and *j*. The attention weights are computed as:


(15) $$\alpha_{ij}^{\left(\ell \right)}={\text{softmax}}_{j}\left(\frac{{\left(W_{q}h_{i}^{\left(\ell +0.5\right)}\right)}^{⊤}\left(W_{k}h_{j}^{\left(\ell +0.5\right)}\right)}{\sqrt{d}}+d_{ij}\right),$$

where $W_{q}$ and $W_{k}$ are learnable projection matrices, *d* denotes the hidden feature dimension.

#### 2.2.4 Gated multi-condition fusion

To incorporate conditional information into the diffusion process, we introduce a gated multi-condition fusion module $f_{\text{fuser}}$, illustrated in Fig. 1d. This module is inspired by the Feature-wise Linear Modulation (FiLM) paradigm (Perez *et al.* 2018), where external conditions modulate the latent representation through learnable scaling and shifting parameters, while the diffusion timestep controls the strength of conditioning via a gating mechanism.

We first compute a timestep-dependent gating scalar (or vector) from the latent representation $g_{t}=\phi_{g}(t)$, where $\phi_{g}(\cdot )$ is a learnable function that produces a gate controlling the overall influence of conditioning at timestep *t*. The condition encoding $c_{t}$ is mapped to FiLM parameters via two learnable projections:


(16) $$\gamma_{t}=\phi_{\gamma }(W_{c}c_{t}),\;\beta_{t}=\phi_{\beta }(W_{c}c_{t}),$$

where $\gamma_{t}$ and $\beta_{t}$ denote feature-wise scaling and shifting coefficients, respectively.

The final fused representation is obtained by applying FiLM modulation to, gated by the timestep-dependent factor:


(17) $${\tilde{c}}_{t}=W_{c}c_{t}\;⊕\;\left(g_{t}\;⊙\;\left(\gamma_{t}\;⊙\;W_{c}c_{t}\;⊕\;\beta_{t}\right)\right),$$

where ⊙ denotes element-wise multiplication and ⊕ denotes element-wise addition.

### 2.3 Implementation details

DesignMaster is trained for 200 epochs with a batch size of 8 using the AdamW optimizer (learning rate $5\times 10^{-5}$, weight decay $10^{-12}$) with a stabilized adaptive learning-rate update, and the model achieving the best performance on the validation set is selected for subsequent testing. The diffusion process uses T=500 steps with a polynomial noise schedule and an L2 denoising objective on atomic coordinates and features. The equivariant backbone consists of 6 layers with hidden size 128, FFN dimension 1024, 32 attention heads, and dropout 0.05, and is initialized from a GEOM-pretrained geometric encoder. All experiments were conducted on a workstation equipped with an NVIDIA GeForce RTX 3090 Ti GPU (24 GB) and an Intel Xeon Platinum 8369B CPU. The full DesignMaster model contains approximately 8.3 million trainable parameters. Training the model on PROTAC-DB required approximately 9–13 ms per sample per epoch on a single GPU, while generating one linker sample with 500 diffusion steps takes on the order of one second on the same hardware.

## 3 Results and discussion

We comprehensively evaluated the proposed framework from multiple perspectives, including model performance, property alignment, and distributional consistency.

### 3.1 Model performance

Table 1 reports the quantitative results on PROTAC-DB 2.0 and 3.0. We evaluate model performance using three metrics, namely Validity, Uniqueness, and Recovery, with their detailed definitions and calculation procedures provided in the Supplementary Material. To assess the uncertainty of the results, we further perform bootstrap analysis and report the corresponding 95% confidence intervals based on 1000 resampling iterations.

**Table 1:** Performance comparison on PROTAC-DB 2.0 (V 2.0) and 3.0 (V 3.0).

Dataset	Model	Validity (%)	Uniqueness (%)	Recovery (%)
V2.0	EDM	91.35 [88.56, 94.94]	48.54 [43.56, 53.02]	35.25 [29.61, 41.53]
	DiffLinker	92.11 [89.83, 95.71]	56.80 [52.95, 59.92]	39.20 [33.84, 44.07]
	DiffPROTACs	93.86 [91.35, 95.72]	68.75 [66.97, 71.41]	43.72 [38.72, 48.79]
	DM	**95.04** [93.50, 96.47]	**71.07** [67.88, 74.11]	**49.92** [45.07, 54.47]
V3.0	EDM	91.72 [88.75, 94.57]	45.10 [40.21, 49.01]	33.80 [27.64, 39.96]
	DiffLinker	92.60 [89.28, 94.92]	54.20 [50.64, 58.06]	40.75 [34.36, 47.14]
	DiffPROTACs	93.96 [91.60, 96.31]	67.74 [63.93, 71.18]	44.91 [39.44, 50.38]
	DM	**95.38** [93.42, 97.12]	**70.22** [66.84, 73.48]	46.09 [40.62, 51.71]
	DM (w/o CE)	90.97 [86.84, 94.24]	66.76 [63.12, 70.34]	**47.01** [41.38, 52.84]
	DM (w/o GF)	92.60 [89.51, 95.91]	68.20 [64.72, 71.58]	46.32 [40.71, 51.96]

Results are reported as mean with 95% bootstrap confidence intervals (DM denotes DesignMaster). The best-performing results are highlighted in bold.

Overall, our model achieves the best or highly competitive performance across all metrics. On PROTAC-DB 2.0, it outperforms all baselines in Validity (95.04%), Uniqueness (71.07%), and Recovery (49.92%). On PROTAC-DB 3.0, our method achieves the highest Validity (95.38%) and Uniqueness (70.22%), and remains highly competitive in Recovery (46.09%). These results demonstrate the effectiveness of DesignMaster across PROTAC datasets of different scales and diversity.

Compared with the baselines, the performance gains can be attributed to both architectural and task-specific design choices. EDM relies on an MLP-based message-passing scheme, which is less expressive than our Graphormer-enhanced equivariant network for capturing complex structural dependencies. DiffLinker is designed as a general-purpose linker generator and does not explicitly model the unique multi-component structure of PROTACs. Although DiffPROTACs adapts diffusion to the PROTAC setting, it does not explicitly encode rich molecular attributes during denoising. In contrast, our framework incorporates conditional molecular feature encoding, enabling more chemically informed generation. The consistently higher Recovery, especially on PROTAC-DB 2.0, highlights the importance of explicitly modeling multi-level molecular properties for faithful linker reconstruction.

The ablation results further confirm the effectiveness of each component. Removing the conditional encoding (w/o CE) degrades Validity and Uniqueness, indicating the necessity of molecular attribute guidance. Similarly, removing the time-gated fusion mechanism (w/o GF) leads to lower Validity and Uniqueness, while Recovery remains comparable to the full model, showing that properly balancing structural and conditional information during denoising is critical for robust generation performance.

### 3.2 Property alignment

We evaluate the alignment between generated and real PROTAC linkers by comparing the distributions of key physicochemical descriptors, including Molecular Weight (MW), Topological Polar Surface Area (TPSA), rotatable bond count (RB), and heavy atom count (HA). As shown in Fig. 2, the generated linkers closely follow the empirical distributions of the reference datasets, with substantial overlap observed for MW and TPSA. Similar consistency is also observed for RB and HA, as shown in Fig. S1. Minor deviations appear in the distribution tails, but the overall distributional shapes are well preserved.

**Figure 2:**
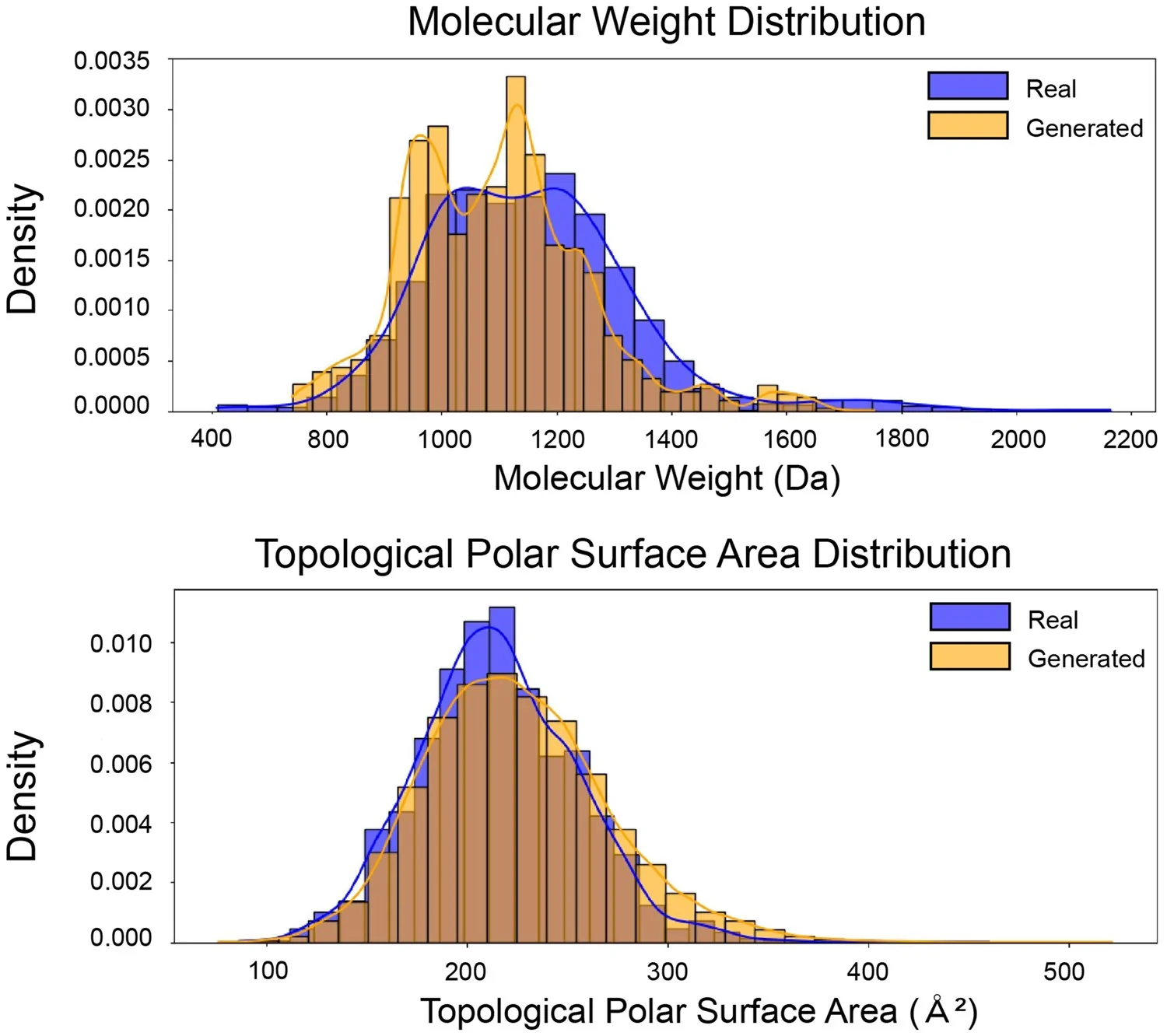
Property distribution gap between generated and true linkers.

To quantify the impact of multi-condition encoding, we compute the Wasserstein distances between the original and reconstructed property distributions, as shown in Fig. 3 and detailed in Table S1. The comparison between the full model and its ablated variant clearly highlights the contribution of conditional modulation.

**Figure 3:**
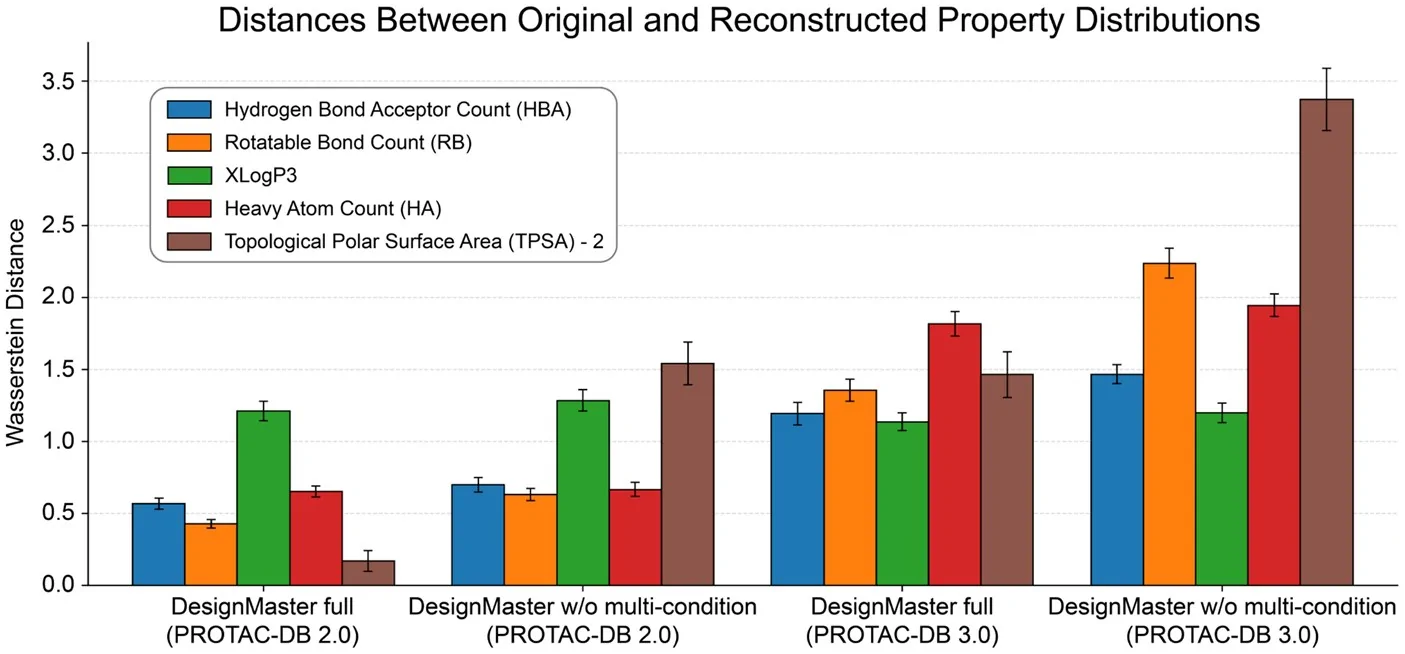
Comparison of Wasserstein distances across DesignMaster variants. Results are reported as the mean over five independent runs, with standard deviations indicated as error bars.

When the multi-condition module is removed (DesignMaster w/o multi-condition), the distribution gaps increase consistently across both PROTAC-DB 2.0 and PROTAC-DB 3.0, particularly for HBA, RB, and TPSA. On PROTAC-DB 2.0, the Wasserstein distances increase from 0.57 to 0.70 for HBA, from 0.43 to 0.63 for RB, and from 2.17 to 3.54 for TPSA after removing multi-condition encoding. A consistent trend is observed on PROTAC-DB 3.0, where the corresponding distances increase from 1.19 to 1.47 for HBA, from 1.36 to 2.24 for RB, and from 3.46 to 5.37 for TPSA. These properties are closely related to hydrogen-bonding capacity, conformational flexibility, and polarity, suggesting that without explicit conditional guidance, the model has greater difficulty preserving the physicochemical characteristics of realistic PROTAC linkers.

For XLogP3 and HA, the differences between the full and ablated models are comparatively smaller, although the full model generally maintains lower distribution gaps. In particular, the Wasserstein distances for XLogP3 change only slightly after removing multi-condition encoding, from 1.21 to 1.28 on PROTAC-DB 2.0 and from 1.14 to 1.20 on PROTAC-DB 3.0. Similarly, the gap for HA increases from 0.65 to 0.67 on PROTAC-DB 2.0 and from 1.82 to 1.95 on PROTAC-DB 3.0. These results suggest that multi-condition encoding has a more pronounced effect on properties related to hydrogen-bonding capacity, conformational flexibility, and polarity, while its influence on molecular size and lipophilicity is comparatively limited.

Overall, both the distribution plots and the Wasserstein distance values demonstrate that our property-aware diffusion framework generates linkers whose physicochemical distributions are better aligned with the true PROTAC linker space, highlighting the effectiveness of explicit molecular attribute encoding.

## 4 Case study

### 4.1 Evaluation of linker reconstruction

To assess the structural validity of generated linkers, we fix the warhead and E3 ligand fragments and generate multiple linker candidates using DesignMaster and DiffPROTACs, respectively. The assembled PROTAC molecules are then subjected to molecular docking against the corresponding ternary complex structures. Docking was performed with AutoDock Vina (Eberhardt *et al.* 2021) using rigid receptors prepared from crystal protein coordinates with ATOM records only and converted to PDBQT format with Open Babel. The search box was centered on the crystallographic PROTAC ligand with 8 Å padding. Each generated full PROTAC was treated as a flexible ligand with exhaustiveness set to 8, and one pose was retained per molecule. Poses were ranked by the heavy-atom RMSD between the docked PROTAC and the crystal conformation, computed in the receptor frame.

For the 6W7O complex, DesignMaster consistently recovers near-native binding geometries, achieving RMSD values of 0.43, 0.56, and 0.64 Å for the top-3 ranked docking poses, compared with 0.65, 1.19, and 1.54 Å for DiffPROTACs (Fig. 4). DesignMaster therefore achieves both lower top-1 RMSD and improved top-*k* stability. Encouraged by this result, we extended the evaluation to a second, structurally distinct ternary complex (PDB: 6HAY) containing the crystallographic PROTAC FX8 (Fig. 5). Beyond RMSD, we performed a more comprehensive analysis including structural overlays and multi-metric analysis. Although the top three poses from both methods exhibit comparably favorable Vina scores (∼−11.5 kcal/mol), DesignMaster achieves a lower mean RMSD than DiffPROTACs (0.78 vs 1.09 Å), together with a higher Morgan Tanimoto similarity (0.88 vs 0.84) and a slightly smaller TPSA. These results indicate that DesignMaster more consistently recapitulates the experimentally observed ternary binding geometry across both complexes, beyond what can be inferred from docking scores alone.

**Figure 4:**
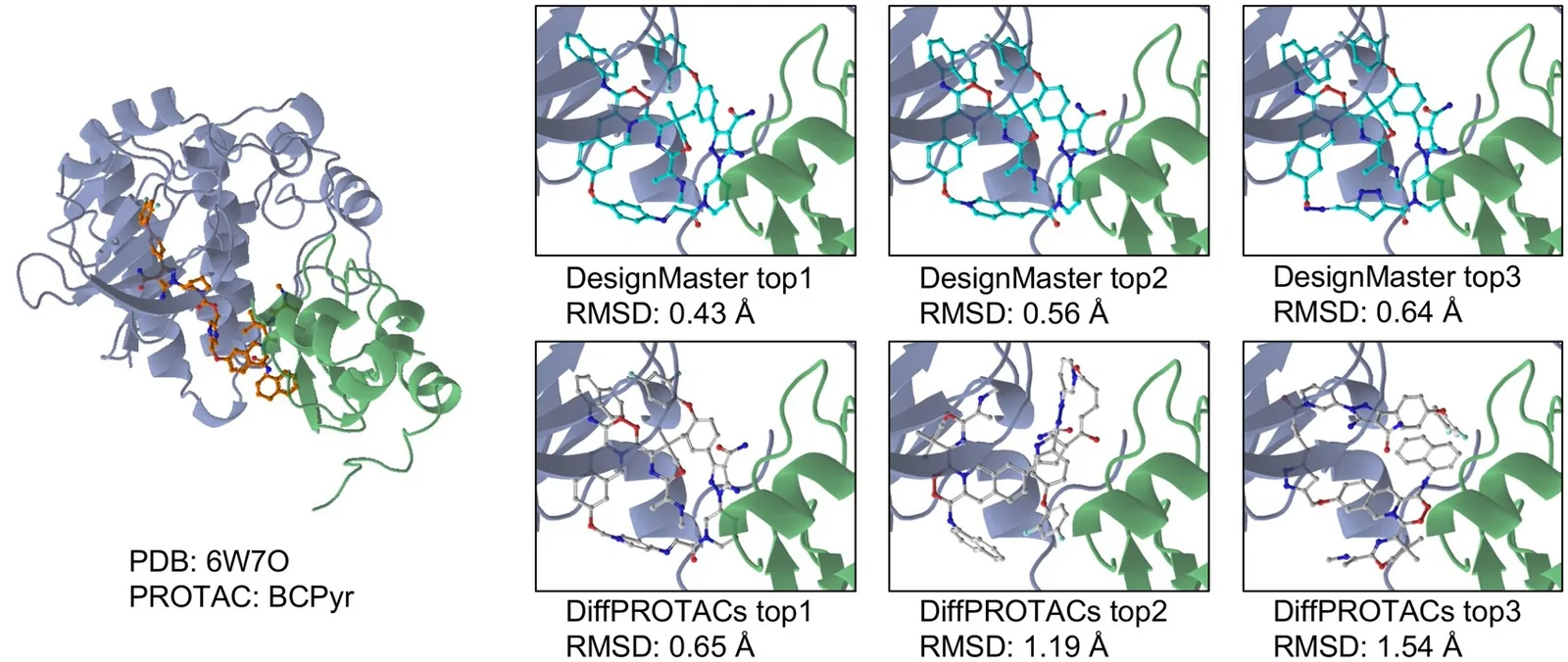
Case study on PDB **6W7O** (PROTAC: **BCPyr**). Top three docked poses from DesignMaster and DiffPROTACs are compared with the reference PROTAC conformation. RMSD is computed to evaluate the structural similarity between the generated and reference PROTACs.

**Figure 5:**
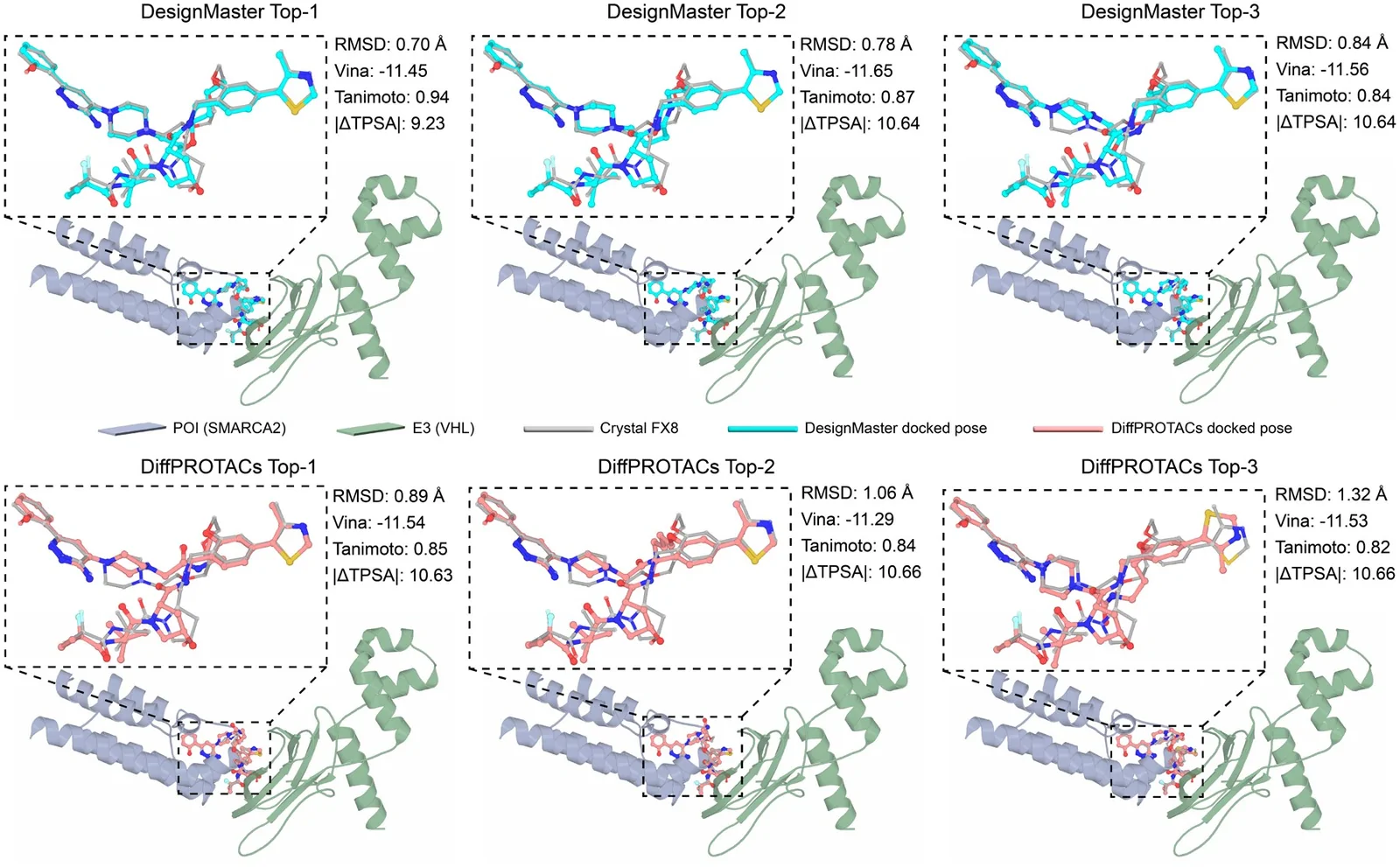
Case study on PDB **6HAY** (PROTAC: **FX8**). Top three docked poses from DesignMaster and DiffPROTACs are overlaid on the co-crystal ligand (gray). Structural and molecular similarity metrics are computed by comparing the generated PROTACs with the reference PROTAC conformation.

We next examined whether the improved geometric agreement was accompanied by preservation of protein–PROTAC interactions. For 6W7O, comparison of the reference and generated complexes (Figs. S5–S7) shows that the DesignMaster-generated complex preserves several interactions observed in the reference structure, including the halogen interaction involving ASP A: 539, the π-alkyl interaction involving VAL A: 416, and the π-cation interaction involving LYS A: 430. Building on this qualitative analysis, we further quantified the recovery of crystal contacts for the 6HAY complex using PLIP (Schake *et al.* 2025). Contacts were classified by interaction type, including hydrogen bonds (HB), hydrophobic interactions (HP), π–π stacking (π–π), water bridges (WB), and halogen bonds (HalB), and the number of crystal contacts recovered at the residue level was summarized in Table 2. DesignMaster poses consistently retained more crystal contacts than DiffPROTACs. Notably, all three DesignMaster poses preserved the crystal water-bridge interaction, whereas none of the DiffPROTACs poses recovered this interaction, π–π stacking and halogen-bond contacts were largely conserved by both methods. A detailed residue-level analysis of the protein–PROTAC interactions was further performed for the DesignMaster Top-1 pose, with conserved, lost, and newly formed interactions summarized in Table S2.

**Table 2:** Comparison of protein-PROTAC interactions between the crystal structure and docked poses.

Model	HB	HP	π -π	WB	HalB	Total

Crystal (FX8)	6	11	2	1	1	21
DesignMaster Top1	5	10	2	1	1	19
DesignMaster Top2	5	8	2	1	1	17
DesignMaster Top3	5	8	2	1	1	17
DiffPROTACs Top1	4	9	2	0	1	16
DiffPROTACs Top2	4	8	2	0	1	15
DiffPROTACs Top3	5	10	2	0	1	18

Together, these results provide complementary structural evidence that DesignMaster generates linker configurations that more faithfully reproduce experimentally observed PROTAC binding geometries and interaction patterns.

## 5 Denoising dynamics analysis

Figure 6 compares the denoising trajectories of DiffPROTACs and our model by tracking total loss, position loss, and feature loss across sampling steps. As only linker atoms are updated during sampling, these losses quantify linker reconstruction throughout the diffusion process. Both methods exhibit a general decreasing trend as the diffusion process progresses, consistent with iterative noise removal. However, our model demonstrates a more stable and consistently downward trajectory, particularly in the later sampling stages. The total loss decreases more smoothly and reaches a lower final magnitude, indicating improved convergence behavior. In contrast, DiffPROTACs shows more pronounced oscillations and a slower decline in the later timesteps, suggesting less stable denoising dynamics.

**Figure 6:**
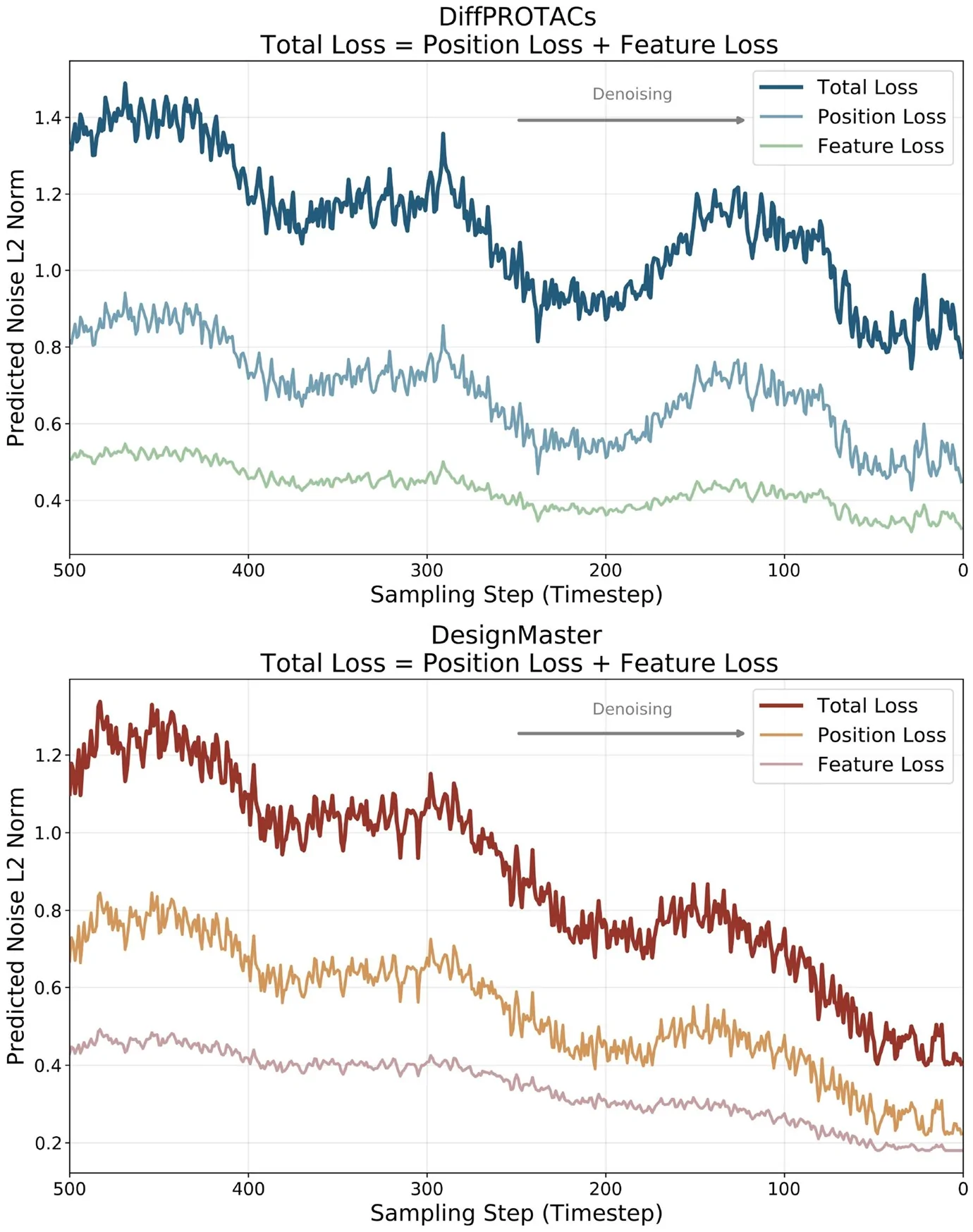
Comparison of linker-level denoising trajectories between DiffPROTACs and DesignMaster, showing total, position, and feature loss trends across time steps.

Notably, the position and feature losses in our model decrease in a coordinated manner, implying that geometric reconstruction and chemical feature optimization progress in a balanced way. This coordinated denoising behavior translates into more stable structural refinement, reducing abrupt conformational shifts during generation. For biological applications, such stability is critical, as it improves reproducibility and reduces the likelihood of generating structurally inconsistent or physically unrealistic linker conformations.

One possible explanation for this improved stability is the incorporation of linker-length and physicochemical constraints, which effectively restrict the sampling space and guide the model toward chemically plausible regions. While these constraints reduce sampling freedom, they act as a beneficial regularization mechanism, preventing excessive structural drift and promoting more reliable convergence.

## 6 Conclusion and discussion

In this work, we propose DesignMaster, a diffusion-based framework for constraint-aware PROTAC linker design. By incorporating linker length and physicochemical properties as conditioning signals within an E(3)-equivariant graph Transformer, the model enables geometry-aware and controllable molecular generation.

Experiments on PROTAC-DB 2.0 and 3.0 show that DesignMaster outperforms existing methods, achieving improved validity and recovery. Docking-based evaluation further demonstrates enhanced structural accuracy, with a 33.85% reduction in RMSD in a case study on IAP-recruiting Bruton’s Tyrosine Kinase (BTK) PROTAC BCPyr (PDB: 6W7O), while an additional case study on the structurally distinct PROTAC FX8 (PDB: 6HAY) further supports the structural fidelity of DesignMaster-generated PROTACs through complementary docking and protein–PROTAC interaction analyses. These results indicate the potential of conditional diffusion modeling for structure-guided PROTAC design.

The current framework treats the warhead and E3 ligand fragments as fixed building blocks, which is consistent with common PROTAC design practices. However, protein-binding pockets may exhibit conformational adaptation in realistic scenarios, and incorporating such flexibility remains an important future direction. Future work could enable limited fragment relaxation through approaches such as rotatable bond sampling or additional conformational optimization strategies to further improve structural realism. Moreover, building on this controllable generative framework, our future studies aim at adaptively reweighting linker property constraints based on the intrinsic physicochemical features of fixed POI and E3 binders, allowing targeted optimization of polarity, hydrogen bonding, and molecular size in a context-dependent manner.

## Funding

F.L. was supported by the Australian National Health and Medical Research Council (NHMRC) Investigator Fellowship (2041439). South Australian immunoGENomics Cancer Institute (SAiGENCI) received grant funding from the Australian Government.

## Supplementary Material

btag714_Supplementary_Data

## Data Availability

The training datasets, pretrained checkpoints, and generated results are publicly available at Zenodo: https://zenodo.org/records/18883030

## References

[btag714-B1] Arnold C. PROTAC protein degraders to drug the undruggable enter phase 3 trials. Nat Med 2024;30:3030–1. 10.1038/d41591-024-00072-8 39379566

[btag714-B2] Axelrod S , Gomez-Bombarelli R. Geom, energy-annotated molecular conformations for property prediction and molecular generation. Sci Data 2022;9:185. 10.1038/s41597-022-01288-4 PMC9023519 35449137

[btag714-B3] Bai N , Miller SA, Andrianov GV et al Rationalizing PROTAC-mediated ternary complex formation using rosetta. J Chem Inf Model 2021;61:1368–82. 10.1021/acs.jcim.0c01451 PMC8866032 33625214

[btag714-B4] Békés M , Langley DR, Crews CM. PROTAC targeted protein degraders: the past is prologue. Nat Rev Drug Discov 2022;21:181–200. 10.1038/s41573-021-00371-6 PMC8765495 35042991

[btag714-B5] Burslem GM , Crews CM. Proteolysis-targeting chimeras as therapeutics and tools for biological discovery. Cell 2020;181:102–14. 10.1016/j.cell.2019.11.031 PMC7319047 31955850

[btag714-B6] Eberhardt J , Santos-Martins D, Tillack AF et al AutoDock Vina 1.2.0: new docking methods, expanded force field, and Python bindings. J Chem Inf Model 2021;61:3891–8. 10.1021/acs.jcim.1c00203 PMC10683950 34278794

[btag714-B7] Ge J , Li S, Weng G et al PROTAC-DB 3.0: an updated database of PROTACs with extended pharmacokinetic parameters. Nucleic Acids Res 2025;53:D1510–5. 10.1093/nar/gkae768 PMC11701630 39225044

[btag714-B8] Hoogeboom E , Satorras VG, Vignac C et al Equivariant diffusion for molecule generation in 3D. In: *International Conference on Machine Learning*, pp. 8867–87. PMLR, 2022.

[btag714-B9] Hornberger KR , Araujo EM. Physicochemical property determinants of oral absorption for PROTAC protein degraders. J Med Chem 2023;66:8281–7. 10.1021/acs.jmedchem.3c00740 PMC10291545 37279490

[btag714-B10] Huang Y , Peng X, Ma J et al 3DLinker: an E(3) equivariant variational autoencoder for molecular linker design. *Proc Mach Learn Res* 2022*;* **162**:9280–94.

[btag714-B11] Igashov I , Stärk H, Vignac C et al Equivariant 3D-conditional diffusion model for molecular linker design. Nat Mach Intell 2024;6:417–27. 10.1038/s42256-024-00815-9

[btag714-B12] Imrie F , Bradley AR, van der Schaar M et al Deep generative models for 3D linker design. J Chem Inf Model 2020;60:1983–95. 10.1021/acs.jcim.9b01120 PMC7189367 32195587

[btag714-B13] Inganas E , Lavén H, Caraballo R et al Conformational dynamics in the cell membrane interactions of bispecific targeted degrader therapeutics. J Med Chem 2025;68:25881–98. 10.1021/acs.jmedchem.5c01499 PMC12750995 41370364

[btag714-B14] Krieger J , Sorrell FJ, Wegener AA et al Systematic potency and property assessment of VHL ligands and implications on PROTAC design. ChemMedChem 2023;18:e202200615. 10.1002/cmdc.202200615 36749883

[btag714-B15] Li B , Ran T, Chen H. 3D based generative PROTAC linker design with reinforcement learning. Brief Bioinform 2023;24:bbad323. 10.1093/bib/bbad323 37670499

[btag714-B16] Li F , Hu Q, Zhang X et al DeepPROTACs is a deep learning-based targeted degradation predictor for PROTACs. Nat Commun 2022a;13:7133. 10.1038/s41467-022-34807-3 PMC9681730 36414666

[btag714-B17] Li F , Hu Q, Zhou Y et al DiffPROTACs is a deep learning-based generator for proteolysis targeting chimeras. Brief Bioinform 2024;25:bbae358. 10.1093/bib/bbae358 PMC11299039 39101502

[btag714-B18] Li X , Pu W, Zheng Q et al Proteolysis-targeting chimeras (PROTACs) in cancer therapy. Mol Cancer 2022b;21:99. 10.1186/s12943-021-01434-3 PMC8996410 35410300

[btag714-B19] Liu J. , Roy M. J., Isbel L., Li F. (2025). Accurate PROTAC-targeted degradation prediction with degrademaster. Bioinformatics 41:i342–51. 10.1093/bioinformatics/btaf191 PMC12261415 40662822

[btag714-B20] Ma N , Bhattacharya S, Muk S et al Frustration in the protein-protein interface plays a central role in the cooperativity of PROTAC ternary complexes. Nat Commun 2025;16:8595. 10.1038/s41467-025-63713-7 PMC12480974 41022846

[btag714-B21] Nori D , Coley CW, Mercado R. De novo PROTAC design using graph-based deep generative models. *NeurIPS 2022 AI for Science: Progress and Promises*, 2022. https://openreview.net/forum?id=pGyp4o9gky0

[btag714-B22] Paiva S-L , Crews CM. Targeted protein degradation: elements of PROTAC design. Curr Opin Chem Biol 2019;50:111–9. 10.1016/j.cbpa.2019.02.022 PMC6930012 31004963

[btag714-B23] Perez E , Strub F, De Vries H et al Film: visual reasoning with a general conditioning layer. In: *Proceedings of the AAAI Conference on Artificial Intelligence*, Vol. 32, 2018.

[btag714-B24] Roy MJ , Winkler S, Hughes SJ et al SPR-measured dissociation kinetics of PROTAC ternary complexes influence target degradation rate. ACS Chem Biol 2019;14:361–8. 10.1021/acschembio.9b00092 PMC6423499 30721025

[btag714-B25] Sakamoto KM , Kim KB, Kumagai A et al PROTACs: chimeric molecules that target proteins to the Skp1–Cullin–F box complex for ubiquitination and degradation. Proc Natl Acad Sci USA 2001;98:8554–9. 10.1073/pnas.141230798 PMC37474 11438690

[btag714-B26] Schake P , Bolz SN, Linnemann K et al PLIP 2025: introducing protein–protein interactions to the protein–ligand interaction profiler. Nucleic Acids Res 2025;53:W463–5. 10.1093/nar/gkaf361 PMC12230730 40347107

[btag714-B27] Schneuing A , Harris C, Du Y et al Structure-based drug design with equivariant diffusion models. Nat Comput Sci 2024;4:899–909. 10.1038/s43588-024-00737-x PMC11659159 39653846

[btag714-B28] Tran NL , Leconte GA, Ferguson FM. Targeted protein degradation: design considerations for PROTAC development. Curr Protoc 2022;2:e611. 10.1002/cpz1.611 PMC9729011 36469581

[btag714-B29] Vemuri S , Bijigiri LP, Gogte S et al Pro-GAT: reconnecting fragmented PROTACs using graph attention transformer. *J Chem Inf Model* 2026;66:11433–44. 10.1021/acs.jcim.6c01049 42734562

[btag714-B30] Weng G , Cai X, Cao D et al PROTAC-DB 2.0: an updated database of PROTACs. Nucleic Acids Res 2023;51:D1367–72. 10.1093/nar/gkac946 PMC9825472 36300631

[btag714-B31] Weng G , Li D, Kang Y et al Integrative modeling of PROTAC-mediated ternary complexes. J Med Chem 2021;64:16271–81. 10.1021/acs.jmedchem.1c01576 34709816

[btag714-B32] Zhao C , Dekker FJ. Novel design strategies to enhance the efficiency of proteolysis targeting chimeras. ACS Pharmacol Transl Sci 2022;5:710–23. 10.1021/acsptsci.2c00089 PMC9469497 36110375

[btag714-B33] Zheng S , Tan Y, Wang Z et al Accelerated rational PROTAC design via deep learning and molecular simulations. Nat Mach Intell 2022;4:739–48. 10.1038/s42256-022-00527-y

[btag714-B34] Zhong G , Chang X, Xie W et al Targeted protein degradation: advances in drug discovery and clinical practice. Signal Transduct Target Ther 2024;9:308. 10.1038/s41392-024-02004-x PMC11539257 39500878

